# Survival rate of pancreatic cancer in Asian countries: A systematic review and meta-analysis

**DOI:** 10.1097/MD.0000000000045104

**Published:** 2025-10-10

**Authors:** Haleh Ghaem, Hamed Safari, Hossein Kargar Jahromi, Mohebat Vali, Hamed Delam

**Affiliations:** a Research Center for Health Sciences, Institute of Health, Epidemiology Department, School of Health, Shiraz University of Medical Sciences, Shiraz, Iran; b Department of Nursing, Larestan University of Medical Sciences, Larestan, Iran; c Research Center for Noncommunicable Disease, Jahrom University of Medical Sciences, Jahrom, Iran; d Department of Epidemiology, School of Health, Shiraz University of Medical Sciences, Shiraz, Iran; e Student Research Committee, Department of Epidemiology, Shiraz University of Medical Sciences, Shiraz, Iran.

**Keywords:** Asia, pancreas, pancreatic neoplasms, survival rate

## Abstract

**Background:**

So far, no comprehensive study has been conducted regarding the survival rate of pancreatic cancer patients in Asia; Therefore, according to the mentioned points, the present study was designed with the aim of conducting a systematic review to calculate the survival rate of pancreatic cancer in Asian countries in 2024.

**Methods:**

This research is a systematic review and meta-analysis. The researchers of this study examined the articles published in 5 international databases including Medline/PubMed, Scopus, Google Scholar, Web of knowledge and ProQuest. Preparation for data analysis was based on content analysis of the Newcastle-Ottawa Quality Assessment Form. Due to the existence of heterogeneity (with a significance level of <0.1 and *I*^2^ >50%), the random effects model was used with the reverse variance method. Meta-regression was also performed for the HDI index. All analyzes were performed by CMA version 2 statistical software.

**Results:**

Finally, 27 articles were selected to enter the meta-analysis model. The 1-year survival rate of pancreatic cancer was estimated to be 27.6% (95% CI: 24.2%–31.2%). The 5-year survival rate for both sexes was 9.7% (95% CI: 8.8%–10.6%). The estimated 5-year survival rates in men and women were 9.3% (95% CI:8.3%–10.4%) and 10.4% (95% CI: 10.2%–10.7%), respectively. The results of the meta-regression model showed that there is a significant relationship between Human Development Index (HDI) and pancreatic cancer survival rate (point estimate of slope: 4.73, standard error: 0.11, *P*-value < .001).

**Conclusion:**

Overall, the 1- and 5-year survival rates for pancreatic cancer in Asian countries were 27.6% and 9.7%, respectively. This rate was lower than in the United States. It seems that the design, implementation, monitoring and planning of integrated diagnostic and treatment services in Asian countries can greatly increase the survival of pancreatic cancer in the coming decades.

## 1. Introduction

Today, pancreatic cancer is one of the most deadly malignant neoplasms in the world and one of the main causes of cancer-related deaths.^[1]^ Adenocarcinoma is the most common type of pancreatic cancer that develops in the exocrine glands of the pancreas and has a very poor prognosis.^[2]^ According to global statistics in 2022, pancreatic cancer is the 14th most common cancer in terms of incidence and the 6th most common in terms of mortality. The number of new infections and deaths were 511,000 and 467,000, respectively, and these rates in countries with a higher Human Development Index (HDI) are about 4 to 5 times that of other countries; the highest incidence rates are seen in Europe, North America, and Australia/New Zealand, although the rates are highest globally in Armenia in West Asia among men and Uruguay in South America among women.^[3]^ Pancreatic cancer is usually asymptomatic in the early stages, and nonspecific symptoms of the disease, including jaundice, weight loss, light-colored stools, abdominal pain, and fatigue, appear gradually as the tumor progresses.^[4,5]^ Currently, there is no effective and definitive method to treat pancreatic cancer, but surgery, chemotherapy and radiotherapy are used to increase survival or relieve the symptoms of patients.^[2,6]^ Surgical removal of the tumor at an early stage is currently the only effective treatment, but unfortunately, pancreatic cancer is usually clinically silent in the early stages and is often diagnosed at an advanced stage.^[7,8]^ Even after applying the treatment plan, due to the recurrence of the disease, the 5-year survival rate of pancreatic cancer is about 2% to 9%.^[9]^ Furthermore, patients who undergo curative surgery with adjuvant chemotherapy still have a very poor 5-year survival rate of about 15% to 20%, and 80% of patients will relapse after tissue removal.^[10]^

The first step in controlling the disease burden related to cancers in any population is to know their status in the population and also collect information about the incidence, survival, type and location of cancers. The studies conducted regarding the survival of pancreatic cancer in Asian countries have reached different results and the population examined in these studies has also been different. So far, no comprehensive study has been conducted regarding the survival rate of pancreatic cancer patients in Asia; Therefore, according to the mentioned points, the present study was designed with the aim of conducting a systematic review to calculate the survival rate of pancreatic cancer in Asian countries in 2024.

## 2. Methods

### 2.1. Study design

This research is a systematic review and meta-analysis, which is a type of secondary study, and in order to do it, primary published studies were used. This study was designed in 2024 and the search time was conducted in June. The method of doing it was based on the items of the Systematic Review and Meta-Analysis (PRISMA) checklist.^[11]^ The protocol of this research has been registered with the ID (CRD42024619445) on the PROSPERO site. Malignant neoplasm of pancreas has C25 code in ICD-10 version 2019.

### 2.2. Search strategy

The researchers of this study examined the articles published in 5 international databases including Medline/PubMed, Scopus, Google Scholar, Web of knowledge and ProQuest. Also, no time and language restrictions were considered for the search.

The selected keywords for international databases were included “Pancreatic cancer,” “Pancreatic neoplasm,” “cancer of Pancreas,” “Cancer of the Pancreas,” “Neoplasms of Pancreas,” “Pancreatic cancer,” “Pancreatic carcinoma,” “Pancreas tumor,” “Survival,” “Survival analysis,” “Survival rate,” “India,” “China,” “Indonesia,” “Pakistan,” “Bangladesh,” “Japan,” “Philippines,” “Vietnam,” “Iran,” “Thailand,” “Myanmar “, “South Korea,” “Iraq,” “Afghanistan,” “Yemen,” “Uzbekistan,” “Malaysia,” “Saudi Arabia,” “Nepal,” “North Korea,” “Syria,” “Sri Lanka,” “Kazakhstan,” “Cambodia,” “Jordan,” “United Arab Emirates,” “ Tajikistan,” “Azerbaijan,” “Laos,” “Turkmenistan,” “Kyrgyzstan,” “Singapore,” “Lebanon,” “Palestine,” “Oman,” “Kuwait,” “Georgia,” “Mongolia,” “Qatar,” “Armenia,” “Bahrain,” “Timor-Leste,” “Cyprus,” “Bhutan,” “Maldives,” “Brunei” and “Asian countries.”

### 2.3. Inclusion and exclusion criteria of primary studies

All observational studies (cross-sectional, case-control, and cohort) that were published until June 1, 2024 and mentioned pancreatic cancer survival in Asian countries and reported at any time and in any language were included in the analysis. Studies that were about other cancers, survival in people who reported regionally disseminated form and metastasis, as well as review studies and meta-analyses and replication studies were excluded from the study. Also, materials that have not been fully published (Gray literature), such as dissertations, conference summaries, conferences, statistics provided by reputable organizations, etc, were included in the study if they had credibility, required quality, and data availability.

### 2.4. Effect size

Survival at 1 and 5 years: percentage of patients who are still alive 1 and 5 years after their diagnosis of pancreatic cancer.^[12]^

### 2.5. Data collection

Collected data was entered into EndNote, X8 software to review and manage documentation results, and duplicate articles were automatically deleted. After the electronic search of the scientific databases, all the collected articles were independently reviewed by 2 researchers in terms of methodological quality based on the standards, and the articles that had severe differences with the minimum critical evaluation indicators of the articles were excluded from the study. Disagreement between the 2 researchers was assigned to a third expert.

### 2.6. Data extraction form

All the final articles included in the study process were extracted data by a checklist that was prepared in advance. This checklist included the author’s name, publication year, study period, study country, patients’ age, survival time in years, HDI, sample size and survival rate by year for each survival. To address the significant influence of age, we specifically extracted and analyzed relative survival rates.

### 2.7. Qualitative assessment

To evaluate the quality of articles, a checklist has been prepared in previous studies. Preparation for data analysis was based on content analysis of the Newcastle-Ottawa Quality Assessment Form. This tool has 3 different sections including selection (4 questions), comparison (1 question) and result (3 questions) and is divided into 3 categories based on the final scores: good (3 or 4 stars in the selection area and 1 or 2 star in the comparison domain and 2 or 3 stars in the outcome/exposure domain), fair (2 stars in the selection domain and 1 or 2 stars in the comparison domain and 2 or 3 stars in the outcome/exposure domain) and poor (0 or 1 star in the domain of selection or 0 stars in the comparison domain or 0 or 1 star in the outcome/exposure domain).^[13]^

### 2.8. Screening studies

The initial search of studies was done by 2 people, and screening of studies, extraction of results, and evaluation of quality control of articles were done separately by 2 people. If there was no agreement between 2 people, the team leader announced the final opinion about that article.

### 2.9. Statistical analysis

Heterogeneity between studies was checked by Cochran test (with a significance level <0.1) and its combination using the *I*^2^ statistic (with a significance level >50%). Due to the existence of heterogeneity (with a significance level of <0.1 and *I*^2^ >50%), the random effects model was used with the reverse variance method. Meta-regression was also performed for the HDI index. All analyzes were performed by CMA version 2 statistical software.

### 2.10. Risk of bias

Random effects model was used to reduce the risk of bias in the studies.^[14]^ Egger diffusion turbidity assessment test was used to assess the risk of diffusion turbidity. Also, in order to determine the effect of each study on the final result, sensitivity analysis was used.

### 2.11. Ethics approval and consent to participate

The present study was the result of research project No. 31646 which was approved by Shiraz University of Medical Sciences with the Code of Ethics IR.SUMS.SCHEANUT.REC.1403.125.

## 3. Results

### 3.1. Study selection

**Figure 1.**
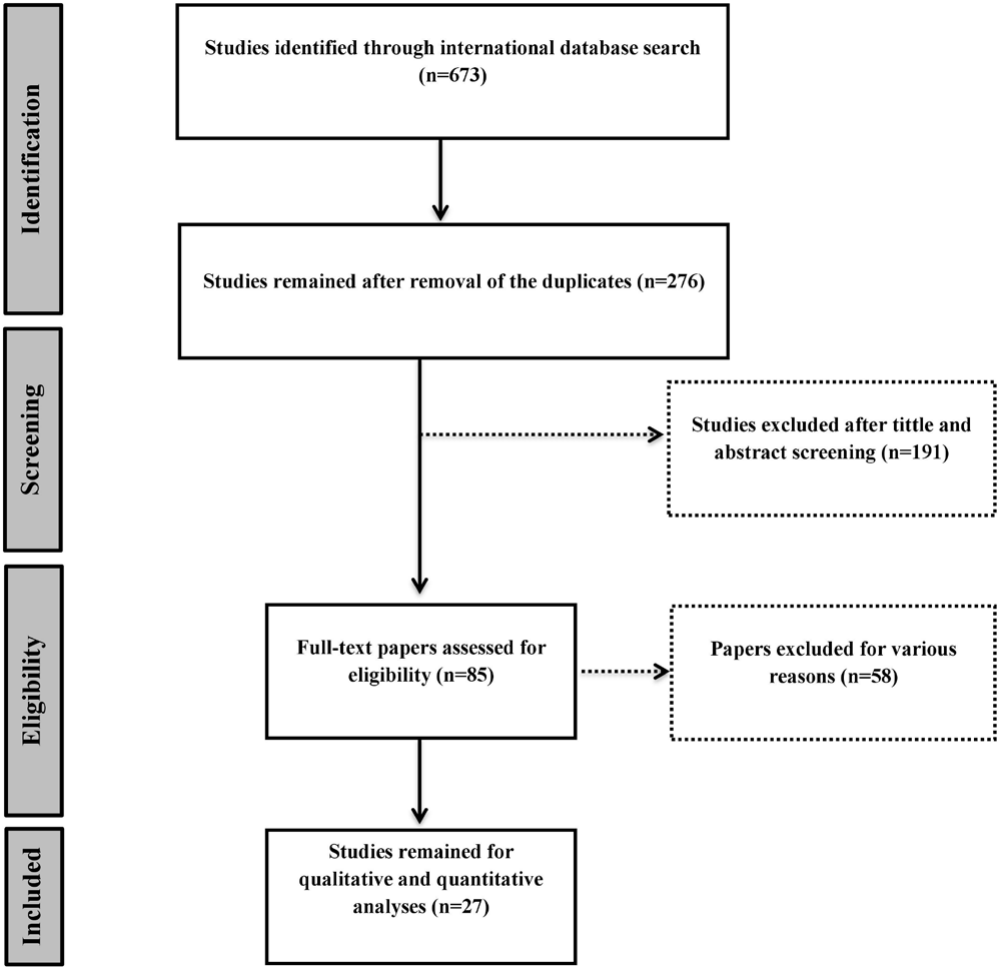
Flow diagram of the study.

At first, a total of 673 articles were obtained from all searched databases. At first, a total of 673 articles were obtained from all searched databases, of which 397 articles were duplicates and were removed. The remaining 276 articles were reviewed for title and abstract. 168 articles were removed due to unrelated objectives and methods. 23 articles did not have full text and 58 articles did not report the considered indicators. Finally, 27 articles were selected to enter the meta-analysis model (Fig. 1). The selected studies were between 2004 and 2023. According to the geographical region, 8 articles from Korea,^[15–22]^ 7 from China,^[23–29]^ 3 from Japan,^[30–32]^ 3 from Iran,^[33–35]^ 2 from Malaysia,^[36,37]^ India,^[38]^ Taiwan,^[39]^ Saudi Arabia^[40]^ and Turkey^[41]^ were included in the model (Table 1).

**Table 1:** Basic information of included studies.

Study name	Survival rate	Survival type	Country	Duration	Gender
Zhang et al^[24]^	54.36%	1	China	1990	Both
Cui et al^[25]^	31.00%	1	China	2019	Both
Eguchi et al^[30]^	28.20%	1	Japan	1981	Both
Eguchi et al^[30]^	34.70%	1	Japan	1981	Both
Chang et al^[39]^	25.52%	1	Taiwan	2002	Both
Chang et al^[39]^	24.17%	1	Taiwan	2002	Male
Chang et al^[39]^	27.34%	1	Taiwan	2002	Female
Luo et al^[26]^	17.80%	1	China	2004	Both
AlGhamdi et al^[40]^	39.00%	1	Saudi Arabia	2000	Both
Ahmadloo et al^[35]^	54.50%	1	Iran	1998	Both
Norsa’adah et al^[36]^	21.40%	1	Malaysia	2001	Both
Tas et al^[41]^	41.00%	1	Turkey	2000	Both
Tas et al^[41]^	13.00%	1	Turkey	2000	Both
Tas et al^[41]^	7.00%	1	Turkey	2000	Both
Yeole and Kumar^[38]^	14.20%	1	India	1987	Both
Li et al^[27]^	37.80%	1	China	2013	Both
Malwinder et al^[37]^	30.80%	1	Malaysia	2002	Both
Jung et al^[22]^	18.30%	1	Korea	1994	Both
Sun et al^[28]^	26.80%	1	China	2013	Both
Sunet al^[28]^	50.00%	1	China	2013	Both
Sun et al^[28]^	53.80%	1	China	2013	Both
Sun et al^[28]^	38.50%	1	China	2013	Both
Sun et al^[28]^	25.60%	1	China	2013	Both
Sun et al^[28]^	17.80%	1	China	2013	Both
Sun et al^[28]^	11.10%	1	China	2013	Both
Vahedi et al^[34]^	29.10%	1	Iran	2016	Both
Tsukuma et al^[31]^	24.20%	1	Japan	1993	Both
Jung et al^[15]^	9.40%	5	Korea	1993	Both
Jung et al^[15]^	7.60%	5	Korea	1996	Both
Jung et al^[15]^	8.00%	5	Korea	2001	Both
Jung et al^[15]^	8.70%	5	Korea	2007	Both
Jung et al^[15]^	8.80%	5	Korea	1993	Male
Jung et al^[15]^	7.30%	5	Korea	1996	Male
Jung et al^[15]^	8.00%	5	Korea	2001	Male
Jung et al^[15]^	8.10%	5	Korea	2007	Male
Jung et al^[15]^	10.10%	5	Korea	1993	Female
Jung et al^[15]^	8.10%	5	Korea	1996	Female
Jung et al^[15]^	8.10%	5	Korea	2001	Female
Jung et al^[15]^	9.50%	5	Korea	2007	Female
Oh et al^[16]^	8.20%	5	Korea	2001	Both
Oh et al^[16]^	9.40%	5	Korea	2008	Both
Oh et al^[16]^	8.20%	5	Korea	2001	Male
Oh et al^[16]^	9.20%	5	Korea	2008	Male
Oh et al^[16]^	9.70%	5	Korea	2008	Female
Jung et al^[17]^	8.20%	5	Korea	2001	Female
Jung et al^[17]^	10.10%	5	Korea	2010	Both
Jung et al^[17]^	9.80%	5	Korea	2010	Male
Jung et al^[17]^	10.50%	5	Korea	2010	Female
Jung et al^[18]^	8.40%	5	Korea	2001	Both
Jung et al^[18]^	8.40%	5	Korea	2006	Both
Jung et al^[18]^	10.80%	5	Korea	2011	Both
Jung et al^[18]^	8.40%	5	Korea	2001	Male
Jung et al^[18]^	8.10%	5	Korea	2006	Male
Jung et al^[18]^	10.30%	5	Korea	2011	Male
Jung et al^[18]^	8.50%	5	Korea	2001	Female
Jung et al^[18]^	8.70%	5	Korea	2006	Female
Jung et al^[18]^	11.50%	5	Korea	2011	Female
Jung et al^[19]^	10.70%	5	Korea	2011	Both
Jung et al^[19]^	11.40%	5	Korea	2012	Both
Jung et al^[19]^	8.20%	5	Korea	2006	Male
Jung et al^[19]^	10.30%	5	Korea	2011	Male
Jung et al^[19]^	11.20%	5	Korea	2012	Male
Jung et al^[19]^	8.40%	5	Korea	2001	Female
Jung et al^[19]^	11.20%	5	Korea	2011	Female
Jung et al^[19]^	11.70%	5	Korea	2012	Female
Kang et al^[20]^	10.60%	5	Korea	1993	Both
Kang et al^[20]^	8.70%	5	Korea	1996	Both
Kang et al^[20]^	8.60%	5	Korea	2006	Both
Kang et al^[20]^	13.90%	5	Korea	2015	Both
Kang et al^[20]^	10.00%	5	Korea	1993	Male
Kang et al^[20]^	8.30%	5	Korea	1996	Male
Kang et al^[20]^	8.30%	5	Korea	2006	Male
Kang et al^[20]^	13.00%	5	Korea	2015	Male
Kang et al^[20]^	11.50%	5	Korea	1993	Female
Kang et al^[20]^	9.30%	5	Korea	1996	Female
Kang et al^[20]^	8.80%	5	Korea	2006	Female
Kang et al^[20]^	14.90%	5	Korea	2015	Female
Kang et al^[21]^	10.90%	5	Korea	2011	Both
Kang et al^[21]^	15.20%	5	Korea	2016	Both
Kang et al^[21]^	14.20%	5	Korea	2016	Male
Kang et al^[21]^	8.90%	5	Korea	2006	Female
Kang et al^[21]^	11.60%	5	Korea	2011	Female
Kang et al^[21]^	16.20%	5	Korea	2016	Female
Jiang et al^[23]^	7.20%	5	China	2012	Both
Jiang et al^[23]^	11.34%	5	China	2014	Both
Zhang et al^[24]^	8.47%	5	China	1990	Both
Eguchi et al^[30]^	7.80%	5	Japan	1981	Both
Eguchi et al^[30]^	7.80%	5	Japan	1981	Both
Chang et al^[39]^	6.60%	5	Taiwan	2002	Both
Chang et al^[39]^	5.95%	5	Taiwan	2002	Male
Chang et al^[39]^	7.47%	5	Taiwan	2002	Female
Luo et al^[26]^	4.10%	5	China	2004	Both
Nemati et al^[33]^	12.20%	5	Iran	2014	Both
AlGhamdi et al^[40]^	10.00%	5	Saudi Arabia	2000	Both
Ahmadloo et al^[35]^	27.00%	5	Iran	1998	Both
Tas et al^[41]^	5.00%	5	Turkey	2000	Both
Yeole and Kumar^[38]^	4.10%	5	India	1987	Both
Yeole and Kumar^[38]^	17.20%	5	India	1987	Both
Yeole and Kumar^[38]^	9.20%	5	India	1987	Both
Yeole and Kumar^[38]^	5.80%	5	India	1987	Both
Yeole and Kumar^[38]^	0.00%	5	India	1987	Both
Yeole and Kumar^[38]^	0.00%	5	India	1987	Both
Yeole and Kumar^[38]^	0.40%	5	India	1987	Both
Yeole and Kumar^[38]^	1.30%	5	India	1987	Both
Yeole and Kumar^[38]^	0.90%	5	India	1987	Both
Li et al^[27]^	10.50%	5	China	2013	Both
Malwinder et al^[37]^	3.70%	5	Malaysia	2002	Both
Sun et al^[28]^	8.70%	5	China	2013	Both
Sun et al^[28]^	25.00%	5	China	2013	Both
Sun et al^[28]^	36.90%	5	China	2013	Both
Sun et al^[28]^	9.60%	5	China	2013	Both
Sun et al^[28]^	4.30%	5	China	2013	Both
Sun et al^[28]^	7.10%	5	China	2013	Both
Sun et al^[28]^	0.00%	5	China	2013	Both
Tsukuma et al^[31]^	6.60%	5	Japan	1993	Both
Lu et al^[29]^	18.90%	5	China	2004	Both
Lu et al^[29]^	14.70%	5	China	2004	Male
Lu et al^[29]^	23.30%	5	China	2004	Female
Lu et al^[29]^	30.30%	5	China	2004	Both
Lu et al^[29]^	23.20%	5	China	2004	Both
Lu et al^[29]^	15.90%	5	China	2004	Both
Lu et al^[29]^	11.20%	5	China	2004	Both
Sato et al^[32]^	6.30%	5	Japan	1990	Both

### 3.2. Quality appraisal

This part was trained by 2 experts and completed separately. Finally, 24 studies had good quality and 3 articles had relatively fair quality (Supplementary File 1, Supplemental Digital Content, https://links.lww.com/MD/Q270).

### 3.3. Results of the meta-analysis model

First, the articles were sorted according to the type of survival reported and then analyzed according to the 1- and 5-year survival rates. Also, the number of articles discussing 2, 3, and 4-year survival rates was very low, and the results of their survival rates did not seem logical.

### 3.4. One-year survival rate

**Figure 2.**
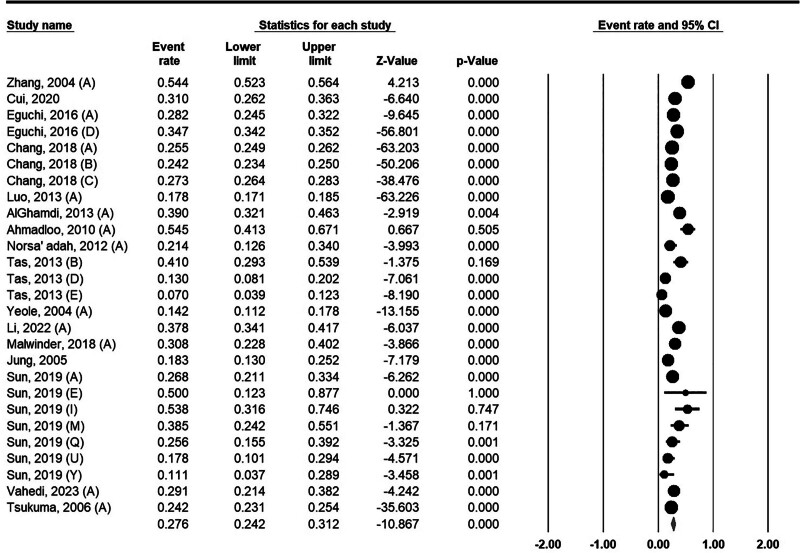
One-year survival rate for pancreatic cancer in Asia.

The 1-year survival rate of pancreatic cancer was estimated to be 27.6% (95% CI: 24.2%–31.2%). The lowest 1-year survival rate was 7% in Turkey^[41]^ and the highest rate was about 54% which was reported in China^[24]^ (Fig. 2).

### 3.5. Overall 5-year survival rate

**Figure 3.**
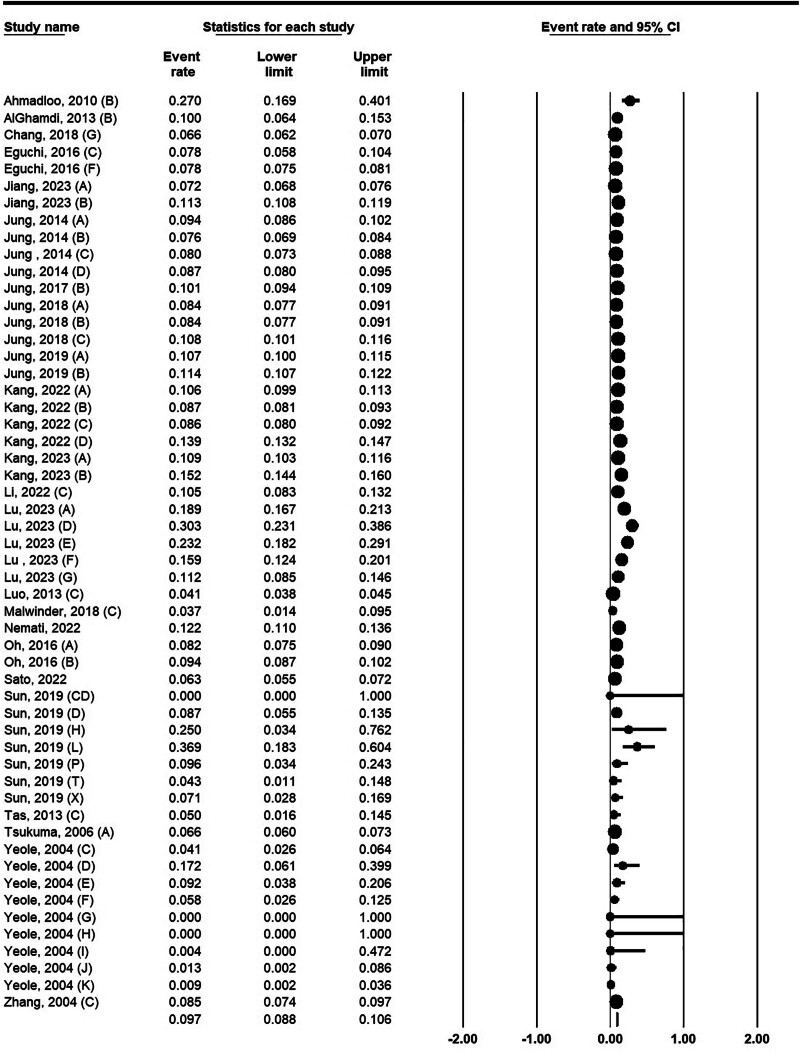
Five-year survival rate for pancreatic cancer in Asia.

The 5-year survival rate for both sexes was 9.7% (95% CI: 8.8%–10.6%). The highest (9.36%) and lowest (0%) survival rates were related to the study of Sun et al.^[28]^ Also, the 5-year survival rate of 0% was reported in Yeole and Kumar’s study^[38]^ (Fig. 3).

### 3.6. The 5-year survival rate by sex

**Figure 4.**
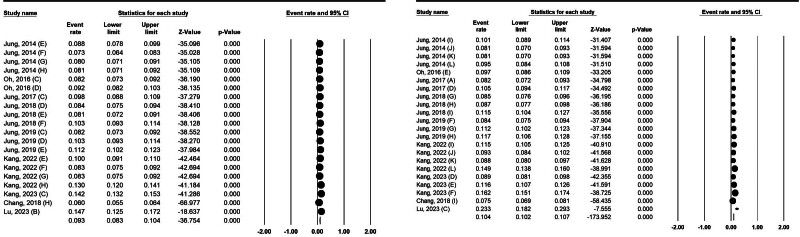
Five-year survival rates for pancreatic cancer in Asia for men (left) and women (right).

The 5-year survival rate in men was estimated at 9.3% (95% CI: 8.3%–10.4%). Lu et al’s^[29]^ study with 14.7% and Chang et al’s^[39]^ study with 6% reported the highest and lowest 5-year survival rates in men, respectively (Fig. 4, left). The 5-year survival rate in women was 10.4% (95% CI: 10.2%–10.7%). Survival rates in women ranged from 7.5% in Taiwan^[39]^ to 23.3% in China^[29]^ (Fig. 4, right).

### 3.7. Meta-regression

**Figure 5.**
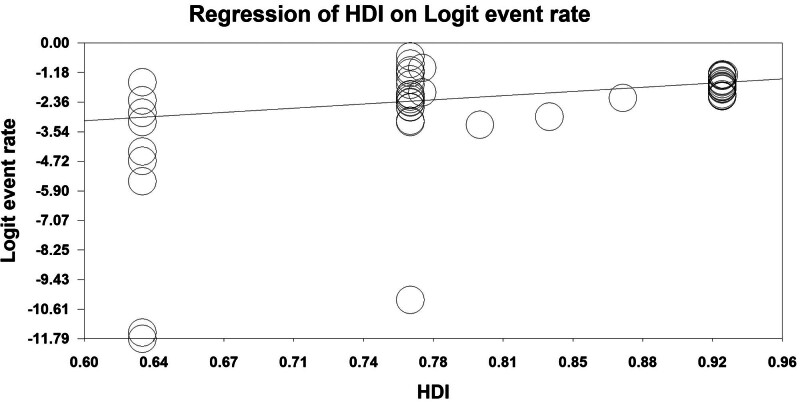
Meta-regression of the association between HDI and pancreatic cancer survival rate. HDI = Human Development Index.

The results of the meta-regression model showed that there is a significant relationship between HDI and pancreatic cancer survival rate (point estimate of slope: 4.73, standard error: 0.11, *P*-value < .001); Thus, countries with higher HDI also reported higher survival rates (Fig. 5).

### 3.8. Risk of bias

**Figure 6.**
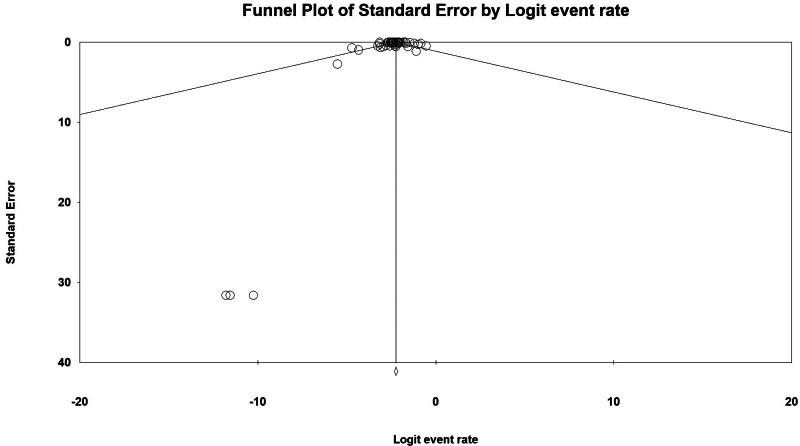
Funnel plot.

Begg and Egger tests were used to estimate the asymmetry of the data. The results of this test showed that there is no publication bias (*P*-value = .749). The funnel plot also showed no publication bias (Fig. 6). Also, sensitivity analysis was performed and the findings showed that there were no outlier results.

## 4. Discussion

In this systematic review and meta-analysis, the 1- and 5-year survival rates of pancreatic cancer in Asian countries were estimated. The results of the present study showed that the 1-year survival rate of pancreatic cancer was about 27.6%. In the study of Lepage et al,^[42]^ which was conducted on cancer patients in 29 European countries, the 1-year survival rate of pancreatic cancer was reported to be 26%. In the study of Bouvier et al,^[43]^ the 1-year survival of pancreatic cancer in the Italian population was 26%, which was almost similar to the results of the present study. In this regard, another study that analyzed data from the Surveillance, Epidemiology and End Results (SEER) registries showed that the 1-year relative survival rate of pancreatic cancer increased from 17% in 1981 to 28.2% in 2010.^[44]^ But another study in the United States found that age-standardized 1-year net survival increased substantially between 2001 to 2003 and 2009 to 2014, from 25.6% to 34.7%.^[45]^

In the present study, it was shown that the 5-year survival rate for both sexes, men and women, is 9.7, 9.3 and 10.4%, respectively. The findings of the study by Sirri et al^[46]^ reported 5-year survival in the American population to be 10.3% and in the German population to be 10.7%. In their study, Exarchakou et al^[47]^ reported that the 5-year survival of British patients increased from 3.6% in 2000 to 2009 to 4.2% in 2010 to 2013. Various studies have also been conducted in Asia. A study based on cancer registry data in South Korea between 1990 and 2019 showed that the 5-year survival rate of pancreatic cancer increased from 8.5% to 13.3%.^[48]^ Another study was conducted in China by Lu et al In this study, the relative 5-year survival rate was evaluated using period analysis and classified based on sex, age at diagnosis and region. In this study, the overall 5-year relative survival rate was estimated to be 18.9%. This rate was 14.7% and 23.3% in men and women, respectively. With increasing age, the survival rate decreased so that the lowest survival rate was observed in more than 74 years.^[29]^ However, another study showed that age and gender do not affect the prognosis of pancreatic cancer patients; but early diagnosis, early radical surgery and chemotherapy can help to improve the prognosis of this disease.^[27]^ One study in the United States showed a 5-year overall survival of 2.5%, which is lower than the results of the present study.^[49]^ But according to the results of the American Cancer Society in 2024,^[4]^ the relative 5-year survival rate of pancreatic cancer in the United States has reached 13% between 2013 and 2019, which is higher than the findings of the present study. Another study showed that after pancreatic cancer was removed, the 5-year survival rate was 34.5%.^[50]^ Tumor characteristics and treatment factors seem to be directly related to survival time in pancreatic cancer patients.^[51]^ It was also shown in a study that the prognosis of pancreatic cancers can be different according to the location of the tumors.^[52]^

In our study, it was shown that there is a significant relationship between HDI and pancreatic cancer survival. Several factors including increasing income, increasing life expectancy, increasing public health, increasing education and awareness of people in different countries can justify this result.^[12]^

### 4.1. Strengths

The present study has several strengths. Data analysis included 27 studies and obtained a very high total sample size. This large-scale analysis provided a high degree of statistical power and enabled a more accurate estimate of the 5-year survival rate in patients with pancreatic cancer. The use of the random effects model led to the findings accurately reflecting the diversity of study populations and methods. The use of the random effects model led to the findings accurately reflecting the diversity of study populations and methods. Subgroup analyzes were performed by gender, which provided insights into different survival outcomes among different subgroups of patients. A further sensitivity analysis confirmed the robustness of our results and showed that our findings are consistent across different methodological approaches.

### 4.2. Limitations

One of the limitations of the present study was the lack of access to the full text of some articles, which may have influenced the overall results of the present study. Also, the reviewed studies did not report the survival rate separately by stage or location of the disease, and inevitably the survival rate was analyzed in general. This study did not report the survival rate separately by treatment modalities, age of the patient, time of diagnosis and tumor size.

## 5. Conclusion

Overall, the 1- and 5-year survival rates for pancreatic cancer in Asian countries were 27.6% and 9.7%, respectively. This rate was lower than in the United States. It seems that the design, implementation, monitoring and planning of integrated diagnostic and treatment services in Asian countries can greatly increase the survival of pancreatic cancer in the coming decades.

## Acknowledgments

The present study with the code of 31646 was approved by the Shiraz University of Medical Sciences. We sincerely thank the Shiraz University of Medical Sciences for its financial support of the study.

## Author contributions

**Conceptualization:** Hamed Delam.

**Data curation:** Hamed Safari, Hossein Kargar Jahromi.

**Formal analysis:** Mohebat Vali.

**Investigation:** Hamed Delam.

**Methodology:** Hamed Delam.

**Supervision:** Haleh Ghaem.

**Validation:** Haleh Ghaem.

**Writing – original draft:** Hamed Delam.

**Writing – review & editing:** Haleh Ghaem, Hamed Delam.

## Supplementary Material


