# Supplementary material for: Survival rate of pancreatic cancer in Asian countries: A systematic review and meta-analysis
Source: Medicine (Baltimore). 2025 Oct 10;104(41):e45104. doi: 10.1097/MD.0000000000045104 (PMC12517833; doi:10.1097/MD.0000000000045104)
Supplement: Supplementary file 1 [file medi-104-e45104-s001.docx]

**Newcastle-Ottawa Quality Assessment Form**

| **Author (year)** | **Selection** | **Comparability** | **Exposure/Outcome** | **Total** | **Quality** |
| --- | --- | --- | --- | --- | --- |
| Zhang QH, 2004 | 3 | 1 | 2 | 6 | Good |
| Cui H, 2020 | 2 | 1 | 2 | 6 | Good |
| Eguchi H, 2016 | 3 | 1 | 2 | 6 | Good |
| Chang JS, 2018 | 3 | 1 | 3 | 7 | Good |
| Luo J, 2013 | 3 | 1 | 3 | 7 | Good |
| AlGhamdi HJ 2013 | 2 | 1 | 3 | 6 | Good |
| Ahmadloo N, 2010 | 2 | 1 | 2 | 5 | Fair |
| Norsa' adah B, 2012 | 3 | 1 | 2 | 6 | Good |
| Tas F, 2013 | 3 | 1 | 3 | 7 | Good |
| Yeole BB, 2004 | 2 | 1 | 2 | 5 | Fair |
| Li Q, 2022 | 3 | 1 | 3 | 7 | Good |
| Malwinder S, 2018 | 3 | 1 | 3 | 7 | Good |
| Jung SW, 2005 | 3 | 1 | 2 | 6 | Good |
| Sun ZX, 2019 | 2 | 1 | 2 | 5 | Fair |
| Vahedi L, 2023 | 3 | 1 | 2 | 6 | Good |
| Tsukuma H, 2006 | 3 | 1 | 2 | 6 | Good |
| Jung K, 2014 | 3 | 1 | 3 | 7 | Good |
| Oh CM, 2016 | 3 | 1 | 3 | 7 | Good |
| Jung KW, 2017 | 3 | 1 | 3 | 7 | Good |
| Jung KW, 2018 | 3 | 1 | 3 | 7 | Good |
| Jung KW, 2019 | 3 | 1 | 3 | 7 | Good |
| Kang MJ, 2022 | 3 | 1 | 2 | 6 | Good |
| Kang MJ, 2023 | 3 | 1 | 2 | 6 | Good |
| Jiang F, 2023 | 3 | 1 | 2 | 6 | Good |
| Nemati S, 2022 | 3 | 1 | 2 | 6 | Good |
| Lu Y, 2023 | 3 | 1 | 2 | 6 | Good |
| Sato Y, 2022 | 3 | 1 | 2 | 6 | Good |
